# Supplementary material for: tiRNA-Val promotes angiogenesis via Sirt1–Hif-1α axis in mice with diabetic retinopathy
Source: Biol Res. 2022 Mar 26;55:14. doi: 10.1186/s40659-022-00381-7 (PMC8962541; doi:10.1186/s40659-022-00381-7)
Supplement: Supplementary file 1 — Additional file 1. Supplementary figures. (a) 1% agarose gel electrophoresis were used to the analysis RNA integrity and the sample with 28S/18S = 1.5–2.0 were chosen for the following research. DR: diabetic retinopathy. (b) From the Northern result of tRNA-Val, we did not find the difference of precursor tRNA. Precursor tRNA containing 5′ leader sequence, 3′ trailer sequence and intron was not considered in this study. High, high glucose. [file 40659_2022_381_MOESM1_ESM.docx]

**Supplementary Figure**

a


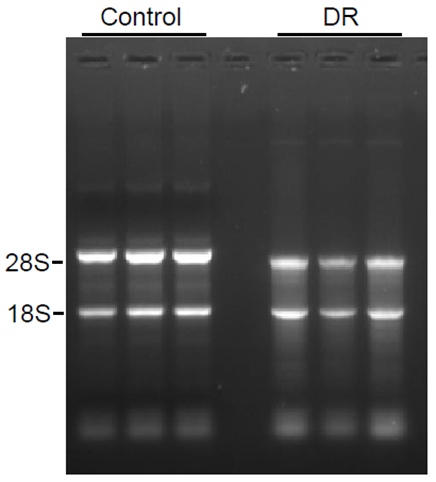


b


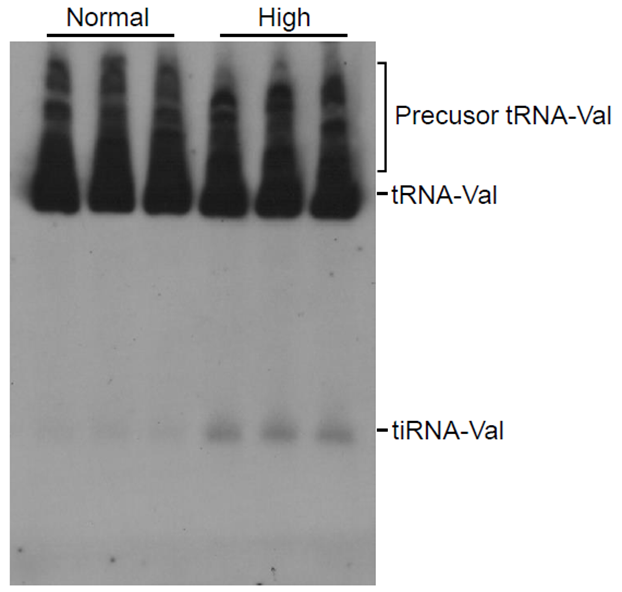


**Supplementary Figure 1**

The RNA/ precursor tRNA sample in this manuscript. (a) 1% agarose gel electrophoresis were used to the analysis RNA integrity and the sample with 28S/18S = 1.5-2.0 were chosen for the following research. DR: diabetic retinopathy (b) From the Northern result of tRNA-Val, we did not find the difference of precursor tRNA. Precursor tRNA containing 5’ leader sequence, 3’ trailer sequence and intron was not considered in this study. High, high glucose.
