# Supplementary material for: tiRNA-Val promotes angiogenesis via Sirt1–Hif-1α axis in mice with diabetic retinopathy
Source: Biol Res. 2022 Mar 26;55:14. doi: 10.1186/s40659-022-00381-7 (PMC8962541; doi:10.1186/s40659-022-00381-7)
Supplement: Supplementary file 2 — Additional file 2. Supporting Information Table. Table S1. Primers for mRNA detection by RT-qPCR. Table S2. Sequences of adapters and primers for tiRNA quantification by TaqMan qRT-PCR. Table S3. 3′-DIG-labelled oligonucleotides probes. [file 40659_2022_381_MOESM2_ESM.docx]

**Supporting Information Table**

**Table S1**

| Primers for mRNA detection by RT-qPCR | |
| --- | --- |
| Name | sequence |
| mβ-actin-FP | GGCTGTATTCCCCTCCATCG |
| mβ-actin-RP | CCAGTTGGTAACAATGCCATGT |
| mVEGF-FP | CTGCTGTAACGATGAAGCCCTG |
| mVEGF-RP | GCTGTAGGAAGCTCATCTCTCC |
| mICAM-1-FP | AAACCAGACCCTGGAACTGCAC |
| mICAM-1-RP | GCCTGGCATTTCAGAGTCTGCT |
| mZO-1-FP | GTTGGTACGGTGCCCTGAAAGA |
| mZO-1-RP | GCTGACAGGTAGGACAGACGAT |
| mANG-FP | GAGCGAATGGAAGCCCTTACAG |
| mANG-RP | GGCAAGCCATTCTCACAGGCAA |
| hβ-actin-FP | CACCATTGGCAATGAGCGGTTC |
| hβ-actin-RP | AGGTCTTTGCGGATGTCCACGT |
| hVEGF-FP | TTGCCTTGCTGCTCTACCTCCA |
| hVEGF-RP | GATGGCAGTAGCTGCGCTGATA |
| hICAM-1-FP | AGCGGCTGACGTGTGCAGTAAT |
| hICAM-1-RP | TCTGAGACCTCTGGCTTCGTCA |
| hZO-1-FP | GTCCAGAATCTCGGAAAAGTGCC |
| hZO-1-RP | CTTTCAGCGCACCATACCAACC |
| hANG-FP | TGGCAACAAGCGCAGCATCAAG |
| hANG-RP | GCAAGTGGTGACCTGGAAAGAAG |

**Table S2**

| Sequences of adapters and primers for tiRNA quantification by TaqMan qRT-PCR | | |
| --- | --- | --- |
| Name | sequence |  |
| 3′-RNA adaptor | /5Phos/GAACACUGCGUUUGCUGGCUUUGAGAGUUCUACAGUCCGACGAUC/3ddC/ | |
| Reverse primer | GATCGTCGGACTGTAGAACTC |  |
|  |  |  |
| Name | TaqMan probe | Forward primer |
| tiRNA-Ala-AGC1 | /5FAMs/GAGCGCGTGCTTAGAACACTGCGTTT/3BHQ1/ | GGGGGTGTAGCTCAGTGGTA |
| tiRNA-Ala-GGC1 | /5FAMs/GGTAGAGTATTTGGAACACTGCGTTT/3BHQ1/ | GGTGATATAGCTCAACTGGT |
| tiRNA-Ala-GGC2 | /5FAMs/GGTAGAGCGCTTGGAACACTGCGTTT/3BHQ1/ | GGGTGTGGCTCAGTGGGTAG |
| tiRNA-Ala-GGC2 | /5FAMs/GAGTGCTTGCTTGGAACACTGCGTTT/3BHQ1/ | GATGTAGCTCAGTTGGTAGA |
| tiRNA-Ala-CGC | /5FAMs/GAGCGCATGCTTCGAACACTGCGTTT/3BHQ1/ | GGGGATGTAGCTCAGTGGTA |
| tiRNA-Ala-TGC | /5FAMs/TGGTAGAGCGCATGAACACTGCGTTT/3BHQ1/ | GGGGGTGTAGCTCAGTGGTA |
| tiRNA-Arg-ACG | /5FAMs/ACGCGTCTGACTAGAACACTGCGTTT/3BHQ1/ | GGGCCAGTGGCGCAATGGAT |
| tiRNA-Arg-CCG | /5FAMs/AGGCGTCTGATTCGAACACTGCGTTT/3BHQ1/ | GGCCGCGTGGCCTAATGGAT |
| tiRNA-Arg-CCT | /5FAMs/AtAAGGCACTGGCGAACACTGCGTTT/3BHQ1/ | GCCCCAGTGGCCTAATGGAT |
| tiRNA-Arg-TCG | /5FAMs/AGGCGTCTGACTTGAACACTGCGTTT/3BHQ1/ | GGCCGCGTGGCCTAATGGAT |
| tiRNA-Arg-TCT1 | /5FAMs/GCGCATTGGACTTGAACACTGCGTTT/3BHQ1/ | GGCTCTGTGGCGCAATGGAT |
| tiRNA-Asn-GTT | /5FAMs/GCGCGTTCGGCTGGAACACTGCGTTT/3BHQ1/ | GTCTCCGTGGCGCAATCGGT |
| tiRNA-Asp-GTC | /5FAMs/GTATCCCCGCCTGGAACACTGCGTTT/3BHQ1/ | TCCTCGTTAGTATAGTGGTT |
| tiRNA-Cys-ACA | /5FAMs/GAGCATTTGACTAGAACACTGCGTTT/3BHQ1/ | GGAGGCATAGCTCAGAGGTA |
| tiRNA-Cys-GCA | /5FAMs/GAGCATTTGACTGGAACACTGCGTTT/3BHQ1/ | GGGGGTATAGCTCAGTGGTA |
| tiRNA-Gln-CTG | /5FAMs/GCACTCTGGACTCGAACACTGCGTTT/3BHQ1/ | GGTTCCATGGTGTAATGGTT |
| tiRNA-Gln-TTG | /5FAMs/GCACTCTGGACTTGAACACTGCGTTT/3BHQ1/ | GGTCCCATGGTGTAATGGTT |
| tiRNA-Glu-CTC1 | /5FAMs/GGATTCGGCGCTCGAACACTGCGTTT/3BHQ1/ | TCCCTGGTGGTCTAGTGGTT |
| tiRNA-Glu-CTC2 | /5FAMs/GAGCATGAGACTCGAACACTGCGTTT/3BHQ1/ | GCCCTGCTAGCTCAGTCGGT |
| tiRNA-Glu-TTC | /5FAMs/GGATTCCTGGTTTGAACACTGCGTTT/3BHQ1/ | TCCCACATGGTCTAGCGGTT |
| tiRNA-Gly-ACC | /5FAMs/GCGCGTTCGCCTAGAACACTGCGTTT/3BHQ1/ | GTTTCCGTAGTGTAGTGGTT |
| tiRNA-Gly-CCC1 | /5FAMs/TCATGCAAGATTCGAACACTGCGTTT/3BHQ1/ | GCGCCGCTGGTGTAGTGGTA |
| tiRNA-Gly-CCC2 | /5FAMs/GAATTCTCGCCTCGAACACTGCGTTT/3BHQ1/ | GCATTGGTAGTTCAATGGTA |
| tiRNA-Gly-GCC | /5FAMs/GAATTCTCGCCTGGAACACTGCGTTT/3BHQ1/ | GCATGGGTGGTTCAGTGGTA |
| tiRNA-Gly-TCC | /5FAMs/GCATAGCTGCCTTGAACACTGCGTTT/3BHQ1/ | GCGTTGGTGGTATAGTGGTG |
| tiRNA-His-ATG | /5FAMs/GAGCGCATGCCTAGAACACTGCGTTT/3BHQ1/ | TGGGGTATAGCTCCATGGTA |
| tiRNA-His-GTG | /5FAMs/GTACTCTGCATTGGAACACTGCGTTT/3BHQ1/ | GCCGAGATCGTATAGTGGTT |
| tiRNA-Ile-GAT | /5FAMs/GAGCATGGTGCTGGAACACTGCGTTT/3BHQ1/ | GGCCAGTTAGCTCAGTTGGT |
| tiRNA-Ile-AAT | /5FAMs/GAGCGTGGTGCTAGAACACTGCGTTT/3BHQ1/ | GGCCGGTTAGCTCAGTTGGT |
| tiRNA-Ile-TAT1 | /5FAMs/GCGCGCGGTACTTGAACACTGCGTTT/3BHQ1/ | GCTCCAGTGGCGCAATCGGT |
| tiRNA-Ile-TAT2 | /5FAMs/TCACATCTGCTTTGAACACTGCGTTT/3BHQ1/ | GGTTCCATAGGGTAGTGGTT |
| tiRNA-Leu-AAG | /5FAMs/AGGCGCTGGATTAGAACACTGCGTTT/3BHQ1/ | GGTAGCGTGGCCGAGCGGTC |
| tiRNA-Leu-CAA | /5FAMs/AGGCGCCAGACTCGAACACTGCGTTT/3BHQ1/ | GTCAGGATGGCCGAGTGGTC |
| tiRNA-Leu-CAG | /5FAMs/AGGCGCTGCGTTCGAACACTGCGTTT/3BHQ1/ | GTCAGGATGGCCGAGCGGTC |
| tiRNA-Leu-TAA | /5FAMs/AGGCGTTGGACTTGAACACTGCGTTT/3BHQ1/ | ACCAGAATGGCCGAGTGGTT |
| tiRNA-Leu-TAG | /5FAMs/AGGCGCTGGATTTGAACACTGCGTTT/3BHQ1/ | GGTAGCGTGGCCGAGCGGTC |
| tiRNA-Lys-CTT | /5FAMs/GAGCGTGGGACTCGAACACTGCGTTT/3BHQ1/ | GCCCAGCTAGCTCAGTTGGT |
| tiRNA-Lys-TTT | /5FAMs/GAGCATCAGACTTGAACACTGCGTTT/3BHQ1/ | GCCCGGATAGCTCAGTCGGT |
| tiRNA-Met-CAT | /5FAMs/GCGCGTCAGTCTCGAACACTGCGTTT/3BHQ1/ | GCCTCGTTAGCGCAGTAGGT |
| tiRNA-Phe-GAA | /5FAMs/GAGCGTTAGACTGGAACACTGCGTTT/3BHQ1/ | GCCGAAATAGCTCAGTTGGG |
| tiRNA-Pro-GGG | /5FAMs/TGGTTCTCGCTTGGAACACTGCGTTT/3BHQ1/ | GGCTTGTTGGTCTGGGGGTA |
| tiRNA-Pro-AGG | /5FAMs/TGATTCTCGCTTAGAACACTGCGTTT/3BHQ1/ | GGCTCGTTGGTCTAGGGGTA |
| tiRNA-Pro-CGG | /5FAMs/TGATTCTCGCTTCGAACACTGCGTTT/3BHQ1/ | GGCTCGTTGGTCTAGGGGTA |
| tiRNA-Pro-TGG | /5FAMs/TGATTCTCGGTTTGAACACTGCGTTT/3BHQ1/ | GGCTCGTTGGTCTAGGGGTA |
| tiRNA-SeC-TCA | /5FAMs/GGGGTGCAGGCTTGAACACTGCGTTT/3BHQ1/ | GCCCGGATGATCCTCAGTGG |
| tiRNA-Ser-GGA | /5FAMs/GAGCATTAGACTGGAACACTGCGTTT/3BHQ1/ | GCTGAAATAGCTCAGTTGGG |
| tiRNA-Ser-AGA | /5FAMs/AGGCGATGGACTAGAACACTGCGTTT/3BHQ1/ | GTAGTCGTGGCCGAGTGGTT |
| tiRNA-Ser-CGA | /5FAMs/AGGCGTTGGACTCGAACACTGCGTTT/3BHQ1/ | GCTGTGATGGCCGAGTGGTT |
| tiRNA-Ser-GCT | /5FAMs/AGGCGATGGACTGGAACACTGCGTTT/3BHQ1/ | GACGAGGTGGCCGAGTGGTT |
| tiRNA-Ser-TGA | /5FAMs/AGGCGTTGGACTTGAACACTGCGTTT/3BHQ1/ | GCAGCGATGGCCGAGTGGTT |
| Sup-TTA | /5FAMs/AGGCCTTGGACTTGAACACTGCGTTT/3BHQ1/ | ACTGGGATGGCTGAGTGGTT |
| tiRNA-Thr-AGT | /5FAMs/AAGCGCCTGTCTAGAACACTGCGTTT/3BHQ1/ | GGCGCCGTGGCTTAGTTGGT |
| tiRNA-Thr-CGT | /5FAMs/AGGCGTCGGTCTCGAACACTGCGTTT/3BHQ1/ | GGCGCGGTGGCCAAGTGGTA |
| tiRNA-Thr-TGT | /5FAMs/AAGCGCCTGTCTTGAACACTGCGTTT/3BHQ1/ | GGCTCCATGGCTTAGTTGGT |
| tiRNA-Tyr-GTA | /5FAMs/GAGCGGAGGACTGGAACACTGCGTTT/3BHQ1/ | CCTTCGATAGCTCAGTTGGT |
| tiRNA-tiRp-CCA | /5FAMs/GCGCGTCTGACTCGAACACTGCGTTT/3BHQ1/ | GACCTCGTGGCGCAATGGTA |
| tiRNA-Val-AAC | /5FAMs/TCACGTTCGCCTAGAACACTGCGTTT/3BHQ1/ | GTTTCCGTAGTGTAGTGGTT |
| tiRNA-Val-CAC | /5FAMs/TCACGCTCGCCTCGAACACTGCGTTT/3BHQ1/ | GTTTCCGTAGTGTAGTGGTT |
| tiRNA-Val-TAC | /5FAMs/TCACGTCTGCTTTGAACACTGCGTTT/3BHQ1/ | GGTTCCATAGTGTAGCGGTT |
| tiRNA-Val-GAC | /5FAMs/GAGTGCTTGCCTGGAACACTGCGTTT/3BHQ1/ | GGGGGTGTGTCTTAGTTGGT |

**Table S3**

| 3'-DIG-labelled oligonucleotides probes | | |
| --- | --- | --- |
| Name | sequence | Modification |
| tiRNA-Val probe | GTGATAACCACTACACTACGGAAAC | 3'-DIG |
